# Synthesis of Clinical-Grade [^18^F]-Fluoroestradiol as a Surrogate PET Biomarker for the Evaluation of Estrogen Receptor-Targeting Therapeutic Drug

**DOI:** 10.1155/2013/278607

**Published:** 2013-05-09

**Authors:** Manish Dixit, Jianfeng Shi, Ling Wei, George Afari, Sibaprasad Bhattacharyya

**Affiliations:** ADRD, SAIC-Frederick, Frederick National Laboratory for Cancer Research, Frederick, USA

## Abstract

16**α**-[^18^F]-fluoroestradiol ([^18^F]FES), a steroid-based positron emission tomography (PET) tracer, has emerged as a dependable tracer for the evaluation and management of estrogen receptor-positive (ER+) breast cancer patients. We have developed a fully automatic, one-pot procedure for the synthesis of [^18^F]FES using the Eckert & Ziegler (E & Z) radiomodular system. After [^18^F]fluorination, the intermediate was hydrolyzed with 2.0 M HCl twice and neutralized with sodium bicarbonate. After high-performance liquid chromatography (HPLC) purification, the decay-corrected radiochemical yield and purity of [^18^F]FES were 40 ± 5.0% (*n* = 12) and >97%, respectively. The product was stable up to 10 h. Total synthesis time including HPLC purification was 80 min. This new, fully automated rapid synthetic procedure provided high and reproducible yields of [^18^F]FES. Quality control (QC) tests showed that the [^18^F]FES produced by this method met all specifications for human injection.

## 1. Introduction 

Fluoroestradiol (FES) has high binding affinity for estrogen receptors and has high tissue permeability including the blood-brain barrier [1, 2]. In clinical setting, using [^18^F]FES as radiopharmaceutical, clear PET images of primary and metastatic breast tumors can be obtained. Prior studies have suggested that [^18^F]FES can be used as a valuable PET tracer to determine the tissue levels of ER in patients with breast cancer and may emerge as a valuable tool to predict which patients with primary, recurrent, or metastatic breast cancer will respond to hormone therapy [3, 4]. In order to validate this potential use of [^18^F]FES, a multicenter clinical trial will eventually be essential, and [^18^F]FES will need to be manufactured at the individual sites. However, routine production of clinical-grade [^18^F]FES presents many challenging laboratory requirements. Yield and product quality may vary from one site to other.

In-vivo target interaction studies of experimental drug in human by PET (using a surrogate tracer) can reduce the substantial uncertainty in early-phase drug development [5]. In our newly established United States Pharmacopeia (USP) laboratory at the Frederick National Laboratory for Cancer Research, we are developing clinical-grade [^18^F]FES as a surrogate biomarker to support the endoxifen clinical trial (Phase I) in breast cancer patients [6, 7]. Endoxifen is an ER-targeting experimental drug related to another U.S Food and Drug Administration-(FDA-) approved ER+ cancer drug named tamoxifen and is under evaluation for the treatment of ER+ cancers. PET scans using [^18^F]FES before (baseline) and after endoxifen treatment can predict the binding efficiency of the drug to the target. The change in [^18^F]FES uptake before and after treatment (using PET) at different endoxifen doses can be useful to assess the effective dose of this drug [5, 7]. 

 We have been using an automatic synthesizer for our USP-grade [^18^F]FES production. The use of automatic synthesizer should minimize the variance in the chemical reaction when compared with manual synthesis. The automated strategy, designed for the development of radiotracers, increases the reliability and reproducibility of a desirable qualitative product and improves the radiation safety. Depending on the type of synthesizer used, reaction conditions and approaches need to be changed to maintain the uniform quality (USP grade) of the product [8–11]. For example, the reaction conditions for [^18^F]FES synthesis in the GE TRACERlab MX_ FDG_ system [9] may not be identical to those needed when other automated synthesizers are used for the same synthesis. 

 The first synthesis of [^18^F]FES was reported by Kiesewetter et al. [12]. In past few years, several research groups have reported [^18^F]FES production using different approaches. However most of these syntheses provide a low, unstable radiochemical yield with unwanted impurities in the product [8, 12, 13]. Therefore, it is a challenge to optimize the production of [^18^F]FES in a new site using a different type of synthesizer without compromising the yield and quality (clinical grade) of the product. The flexibility of the commercially available E & Z Modular Lab platform has prompted us to develop an automated procedure for the routine synthesis of clinical-grade [^18^F]FES with this system.

 Herein, we describe a fully automated one-pot synthesis of clinical-grade [^18^F]FES using the E & Z Modular Lab synthesizer that results in a high radiochemical yield. QC data from three qualification runs are provided to show that the product is indeed a USP grade sufficient for human administration. 

## 2. Materials and Methods

### 2.1. Modular-Lab System

The individual modules, used to build this “modular-lab” system, were purchased from E & Z Eurotope GmbH (Berlin, Germany).

**Figure 1:**
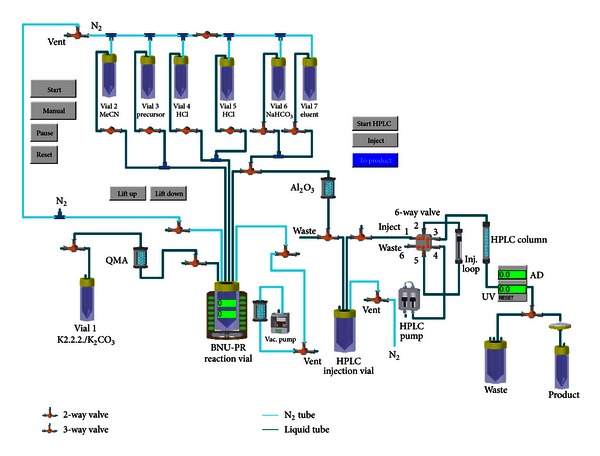
Schematic diagram of the automated synthesis of [^18^F]FES for medical use.

 The E & Z system was configured according to a [^18^F]FES synthesis sequence program (Figure 1) developed by us. In this configuration, one Peltier reactor module (PRM), which allows temperature control from −40°C to +150°C, was used. This reactor is equipped with a magnetic stirrer, temperature and radioactivity sensors, a pneumatic reactor lift, and a reactor camera. The reactions were carried out in a 10 mL borosilicate glass V-vial (Alltech) equipped with reactor head from E & Z. A [^18^F]-fluoride-trapped QMA cartridge obtained from the cyclotron facility was connected to the line between the Kryptofix 2.2.2 (K2.2.2) vial (vial 1) and the reaction vial (BNU-PR). Activity was eluted from the cartridge to the reaction vial under vacuum generated by vacuum pump attached to the reactor through a liquid nitrogen trap. All other reagents (liquids) in vials 2 through 7 were transferred to the reaction vial under a positive pressure of nitrogen. Transfers were controlled by the automatic switching of the in-line two-way and three-way valves on the modules. Azeotropic drying of [^18^F]-fluoride was done under vacuum with a constant flow of nitrogen regulated by a flow controller module. After reaction, the crude product in the reaction vial was transferred to an HPLC injection vial through an alumina cartridge using the pneumatic lift, which delivered the transfer line to the bottom of the reaction vial at the time of the transfer process. HPLC purification was performed on a preparative column (Zorbax SB-phenyl 9.4 × 250 mm, Agilent) integrated into an HPLC module equipped with a six-port-multichannel valve, a fluid sensor, a preparative sample loop, a radioactive detector, a fixed-wavelength (*λ* = 254 nm) UV detector, and an HPLC pump. The complete system, along with a cold trap and vacuum pump, was placed inside a lead-shielded hood, whereas the programmable logic controller, and the cooling system were located under the hood. All processes were remotely controlled by a computer employing the dedicated modular-lab software interface from E & Z, which allowed setup of the interactive process scheme, flexible programming and provides USP/GMP-compliant batch records including temperature, activity, and UV traces. 

After the complete decay of radioactivity, the system was cleaned by filling the reagent vials with sterile water, then acetone, and lastly by a stream of nitrogen using an automated program (clean cycle) that is similar to the [^18^F]FES synthesis program, but without the heating and stirring steps. The reaction vial and precursor vial were replaced by cleaned and oven-dried vials every time before starting a new [^18^F]FES batch production.

### 2.2. Reagents, Solvents, and Disposables

The precursor, 3-methoxymethyl-16*β*, 17*β*-epiestriol-O-cyclic sulfone (MMSE), and authentic nonradioactive standard [^19^F]FES were obtained from ABX, Germany. Solvents and reagents were purchased from Sigma (Milwaukee, WI, USA) and were used without further purification. Sterile vial, USP-grade 0.9% NaCl, and sterile water for injection were purchased from Hospira. Since our Frederick campus does not have a cyclotron yet, [^18^F]-fluoride trapped in a QMA cartridge was purchased from Cardinal Health, Beltsville, MD, USA, or from PETNET, Philadelphia, PA, USA. The Sep-Pak Alumina light cartridge was purchased from Waters Corporation and was flushed with 10 mL of ethanol, followed by 10 mL of sterile water, prior to use.

### 2.3. Radiosynthesis

**Figure 2:**
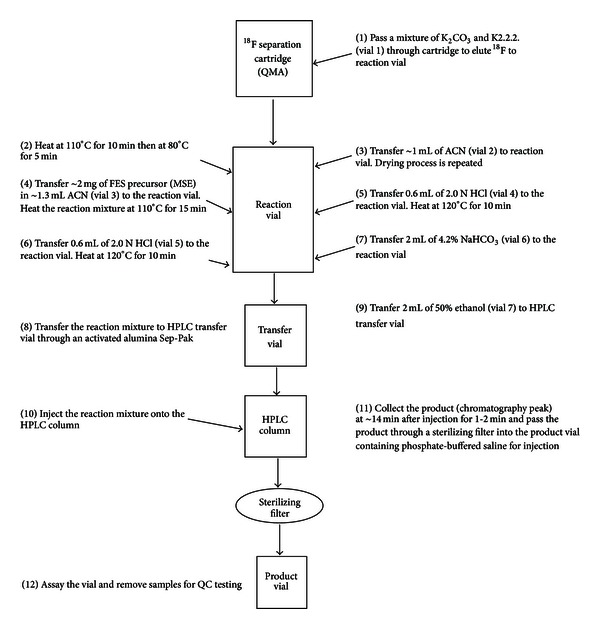
Schematic flow chart of the process for radiosynthesis of [^18^F]FES.

The [^18^F]-fluoride was eluted into the reaction vial from the ^18^F-trapped QMA cartridge with 1 mL of solution prepared by mixing of 100 *μ*L of 0.25 M K_2_CO_3_ and 900 *μ*L of K2.2.2 (15 mg/mL in MeCN). The precursor, MMSE (1.0 mg) in anhydrous MeCN (1.2 mL), was added to the azeotropically dried K2.2.2/K[^18^F]F, and the mixture was heated at 110°C for 15 min. The solution was then hydrolyzed in the same pot by heating the reaction mixture with 2.0 M HCl (0.6 mL) for 10 min at 120°C. The hydrolysis process was repeated after addition of fresh 2.0 M HCl (0.6 mL) to the reaction mixture. The reaction mixture was then neutralized by adding 2.0 mL of 4.2% NaHCO_3_ (USP grade). The crude product was passed through an activated alumina Sep-Pak to an HPLC injection vial where it was diluted with 2.0 mL of 50% ethanol (HPLC mobile phase). This solution was then loaded onto an HPLC loop for the separation of [^18^F]FES. The semipreparative HPLC module equipped with a semipreparative column (Zorbax-SB-Phenyl, Agilent), a UV detector (fixed *λ* = 254), and a radiation detector was used to purify the crude product at a flow rate of 3 mL/min using 50% ethanol in injectable water as the mobile phase. The [^18^F]FES peak was collected in a vented sterile vial filled with formulation buffer (~25 mL 0.15 M phosphate, USP) through a 0.22 micron (*μ*m) filter. The general procedure for the synthesis of [^18^F]FES is shown as a flow chart in Figure 2.

### 2.4. QC Procedures

 QC of [^18^F]FES prepared at our laboratory for clinical use is carried out according to the USP recommendations detailed below. After successfully meeting all release criteria, doses are released to physicians for clinical use. 

#### 2.4.1. Particulates. 

The [^18^F]FES product solution is examined visually. Evaluating the chemical purity by visual inspection is straightforward.The final drug product in the vial should be clear and colorless without any visible particulates as per USP 〈823〉 and USP 〈631〉 Color and Achromaticity. 

#### 2.4.2. Filter Integrity Test

Because the USP sterility test requires 14 days to complete, the [^18^F]FES product solution sterility cannot be assured prior to injection. The [^18^F]FES product is passed through a 0.22 *µ*m sterilizing filter into the final product vial. After the [^18^F]FES product is collected, the sterilizing filter is tested for filter integrity to give an indication of likelihood of the product sterility. Filter integrity is tested in a bubble point procedure, whereby the sterilizing filter is placed on a gas line with a pressure gauge and the outlet of the filter is placed under water. The gas pressure on the inlet to the filter is increased slowly until a steady stream of bubbles is observed at the filter outlet. The pressure at which the bubble stream begins is recorded and compared with the manufacturer's pressure rating (typically 50 psi) for the filter (from the certificate of quality). If the initial integrity test fails filter will be rewetted with 30 mL of water and retested.

#### 2.4.3. Kryptofix [2.2.2] Test

The qualified K2.2.2 test is based on the method by Mock et al. [14], which uses a color spot test for the detection of residual K2.2.2 in the final drug product. The FDA has proposed a maximum permissible level of 50 *µ*g/mL of K2.2.2 in 2-[^18^F]-fluoro-deoxy-glucose; therefore this maximum permissible level is appropriate for the [^18^F]FES final product.

#### 2.4.4. Chemical Purity and Radiochemical Purity/Identity

Analytical HPLC analysis for the QC of the final tracer product was carried out on a Shimadzu HPLC system equipped with a variable UV detector preset to 280 nm and a radioactive detector (Carroll and Ramsey Associates, CA, USA). The samples were injected on to an analytical C18 column (Phenomenex Gemini, 4.6 × 150 mm, 110 A, 5 *μ*m), which was eluted with a mobile phase of 50% ethanol : 50% water (v : v). The column flow rate is 0.5 mL/min and was kept at approximately room temperature, 25 to 30°C. The typical retention of FES is somewhere in between 9.2 and 9.5 min for the UV absorbance (the radioactivity detector is ~0.2 min further downstream from the UV detector). The standard concentrations must bracket the sample or bracket the minimum acceptable mass limit. All standards must be baseline resolved (resolution > 1.5) for a valid analysis. A linear regression is determined for UV absorbance peak areas of the standards. This constitutes the calibration curve. Then the UV peak area of the FES drug product is fit on the calibration curve to determine the FES concentration in the drug product sample. The amounts of UV-impurities are measured using the same standard calibration curve. 

The use of radio-thin-layer chromatography (TLC) to determine the radiochemical purity and identity was validated using HPLC-grade methanol as the mobile phase. The *R*_*f*_ values obtained from these studies were between 0.70 and 0.80 for the three qualification runs. The limit was set for *R*_*f*_>0.5 for the final [^18^F]FES because the TLC test was included only to separate unbound fluoride (origin) from the product (solvent front). The specification for the purity is greater than or equal to 95% using this methodology. This is primarily a test for free fluoride. If present, unlabeled ^18^F will remain at the origin with an *R*_*f*_ value equal to 0 and so is an adjunct test to the analytical HPLC. The identity is confirmed by HPLC co-injection of the nonradioactive FES authentic standard with the drug product to confirm that the retention time values are consistent.

#### 2.4.5. Radionuclidic Identity

Radionuclidic identity is confirmed by measuring the half-life of radiopharmaceutical doses and comparing it to the known half-life of fluorine-18 (109.77 min). For the test, an aliquot of the [^18^F]FES product was counted in an ion chamber or gamma counter at least five times. The half-life of the radioactivity was determined for each activity measurement using the following equation (1):

(1) $$\begin{array}{c} T_{1/2}=-\ln2\left(\frac{\text{time}\;\text{difference}}{\left(\ln\left(\text{ending}\;\text{activity}/\text{starting}\;\text{activity}\right)\right)}\right). \end{array}$$

#### 2.4.6. Residual Solvent Analysis

Residual solvent (acetonitrile and acetone) levels in the doses were determined using a Shimadzu GC-2010 with an autoinjector, a flame ionization detector, and a Restek Rtx-Wax column using a method similar to that reported by Channing et al. [15]. The detector signal-to-noise ratio must be greater than 10 : 1 for the maximum allowable concentration for each solvent when injecting 0.5 *μ*L of the analyte. The methods to determine acetone and acetonitrile levels are consistent with or more stringent than USP 〈467〉 Organic Volatile Impurities. Five prepared concentrations of the external standard were analyzed using the gas chromatography (GC) method to obtain values for calculating the standard curve. The retention times were approximately 4.1 minutes for acetone (*k*′ ~ 0.5) and 6.7 minutes for acetonitrile (*k*′ ~ 1.9). All of the peaks for these compounds were baseline resolved. 

#### 2.4.7. Bacterial Endotoxin

Levels of bacterial endotoxin were tested and qualified using one of two procedural methods. Both are based on USP recommendations but use control standard endotoxin referenced to the USP. Either a gel-clot method or the portable test system (PTS) from Charles River Laboratories was used. All of the bacterial endotoxin levels were <175 EU per batch for the initial qualification syntheses. 

#### 2.4.8. pH

Because the product volume is small, as well as radioactive, pH was measured using an appropriate variation of USP 〈791〉 pH. Instead of a pH meter, pH test strips were used. The pH test strips were checked by pipetting pH 5 and pH 7-calibrated commercial pH standards onto individual strips. The color on the strips must match the pH 5 and pH 7 on the color key supplied with the test strips. Then the [^18^F]FES was pipetted onto another test strip, the color checked against the color key, and the result recorded. The measured pH must be between pH 6 and pH 8 for the [^18^F]FES product to be released.

#### 2.4.9. Sterility

Sterility was tested using the direct inoculation method as is required by USP. It is not a releasing test. This test requires ~14 days of time. 

#### 2.4.10. Stability and Expiration Dating

The final drug product was left at room temperature for up to 12 h. During this time, the product was measured periodically for radiochemical purity using analytical HPLC and TLC. In addition, the [^18^F]FES product was examined for changes in UV absorbance of the product peak with time. There was no UV-detectable breakdown of the product over that time period. The expiration time (typically 8–10 hrs) was set based on stability data.

## 3. Results and Discussions

### 3.1. Radiochemistry and Formulation

**Figure 3:**
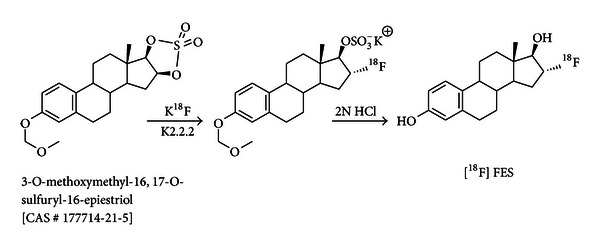
Schematic representation of [^18^F]-fluoroestradiol synthesis.

In a typical procedure using the E & Z automated radiomodular system [^18^F]-fluoride trapped in a QMA cartridge (~200 mCi) was attached in between vial 1 (K2.2.2/K_2_CO_3_) and the reaction vial (BNU-PR). An aqueous K2.2.2/K_2_CO_3_ mixture in acetonitrile was used to elute the [^18^F]-fluoride into the reaction vessel, then the reaction was heated through two temperature steps, 110°C for 10 min and 80°C for 5 min, to azeotropically remove the water and the acetonitrile. A 1 mL volume of acetonitrile was added and the drying process was repeated. Next, the organic precursor for the reaction, MMSE (1-2 mg), in acetonitrile was added to the reaction vessel. The reaction was heated to 110°C for 15 min in a closed reaction vessel to promote the substitution of the ^18^F for the sulfate at the 16 positions of the five-membered carbon ring (Figure 3). Next, 0.6 mL of 2.0 N HCl was added to the same vessel and the reaction was heated to 120°C for 10 min to remove the sulfate- and methylmethoxy-protecting groups from the FES. Hydrolysis was repeated again by addition of 0.6 mL of 2.0 N HCl. A 2 mL volume of 4.2% sodium bicarbonate was added to neutralize the reaction mixture which was then transferred to an HPLC transfer vial through the alumina Sep-Pak to reduce the free [^18^F]-fluoride concentration. Approximately 2 mL of HPLC mobile phase was added to the HPLC transfer vial prior to loading it onto the preparative HPLC column for purification. The resulting [^18^F]FES was separated from the other compounds in the reaction mixture using preparative HPLC. A sterile mobile phase consisting of 50% water : 50% ethanol (v : v) was the preparative mobile phase that was used to elute the purified [^18^F]FES. The radiosynthesis took 80 min from the time that the [^18^F]-fluoride was delivered to the reaction vessel of the automation unit to the time the automation program ended, including HPLC purification. The decay-corrected radiochemical yield for the qualification runs was 40.0 ± 5.0% (*N* = 12).

Although ^18^F-fluorination method is straightforward, effective hydrolysis of the fluorinated intermediate is very challenging. Fluorination methods are almost similar in the literature, but the hydrolysis procedures are quite different, largely depending on the type of synthesizers used. We tried single hydrolysis using the same (2.0 N) and different HCl concentrations. The overall radiochemical yield of [^18^F]FES was 15–20% (corrected, *n* = 4). In semipreparative HPLC, we observed a large peak of radiolabeled side product in between 5 and 8 min. This could be the partially hydrolyzed and/or unhydrolyzed product. We also tried hydrolysis using concentrated H_2_SO_4_ in ethanol as described in the recent literature [11] but we always observed a significant amount of polar, radiolabeled side product (*t*_*R*_ = 6.0 min) in semipreparative HPLC with an unknown structure. Performing double hydrolysis with 2.0 M HCL was very effective in minimizing this radiolabeled side product, with an increase in the overall decay-corrected yield to 40.0 ± 5.0% (*N* = 12). Oh et al. [9] reported that repeated (three times) and evaporative hydrolysis under negative pressure (vacuum) in the reaction vial was very effective in minimizing side products. In our system, evaporative hydrolysis caused significant loss of radioactivity in the reaction vial. Our simple modified two-step, nonevaporative hydrolysis method, using the E & Z system worked very well to minimize the side product and to increase the overall yield. 

**Figure 4:**
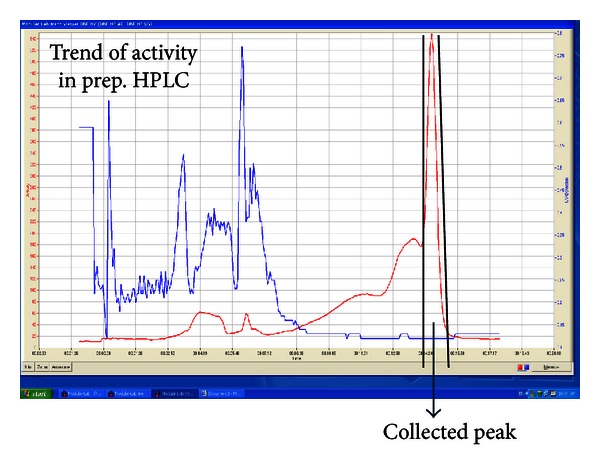
Chromatogram of preparative HPLC separation of [^18^F]FES reaction mixture. Red-colored trace is radioactivity and blue-colored trace is UV absorbance at 254 nm. The two vertical solid lines in the trace show the start (leftmost, ~14 min) and end (right, ~15 min) of the product collection. Earlier broad radiation peak is due to the close vicinity of radiation detector to the HPLC column. Detector is sensing radiation while the compound is moving inside the column.

The product, [^18^F]FES, is eluted around 14 min (42 mL). A typical preparative chromatogram is presented in Figure 4. Most probably, the earlier broad radiation peaks (10 to 14 min) are due to the close vicinity of radiation detector to the HPLC column. The radiation detector is sensing radiation while the compound is moving inside the column. These peaks were collected separately and the radioactivity associated with this peak was measured and found to be very negligible (few *μ*Ci). 

Increasing the amount of precursor (>2 mg) did not increase the radiochemical yield significantly but did increase the level of UV impurities in final product.

The drug substance ([^18^F]FES) is not isolated. Instead, it is collected directly through a 0.22 *µ*m sterilizing filter into a vented sterile vial containing 15 mL of saline for injection, preservative free USP, 10 mL of sterile water for injection USP, and 0.75 mL of sodium phosphate for dilution USP (150 mmol phosphate, 200 mmol sodium per 50 mL) to reduce the ethanol concentration to <15%, bring the pH to 7, and make the final drug product isotonic. Samples are then removed for analysis of product quality. 

### 3.2. QC of the Product

The drug product is assayed for total radioactivity and is examined for particulates. The integrity of the sterilizing filter is tested. Two samples, totaling at least 1.5 mL, are removed for the measurement of pH and residual levels of K2.2.2; for analytical HPLC measurements for specific activity (SA), radiochemical, and chemical purity; for GC measurements of residual solvents; for radionuclidic purity by half-life determination; for apyrogenicity by PTS; and for sterility determination of the product. At least 0.5 mL of the sample is retained for further testing, if necessary. The product dose is drawn, labeled, and once all but the sterility tests have been passed, the product dose is released for injection. A PTS chromogenic endotoxin test must be completed before release of the product. The sample for sterility testing is inoculated within 48 hours but after the sample has decayed to a background radiation level. QC results of three of the completed qualification [^18^F]FES radiosyntheses are listed in Table 1. 

#### 3.2.1. Radiochemical Purity, Dose Amount, and Specific Activity

**Figure 5:**
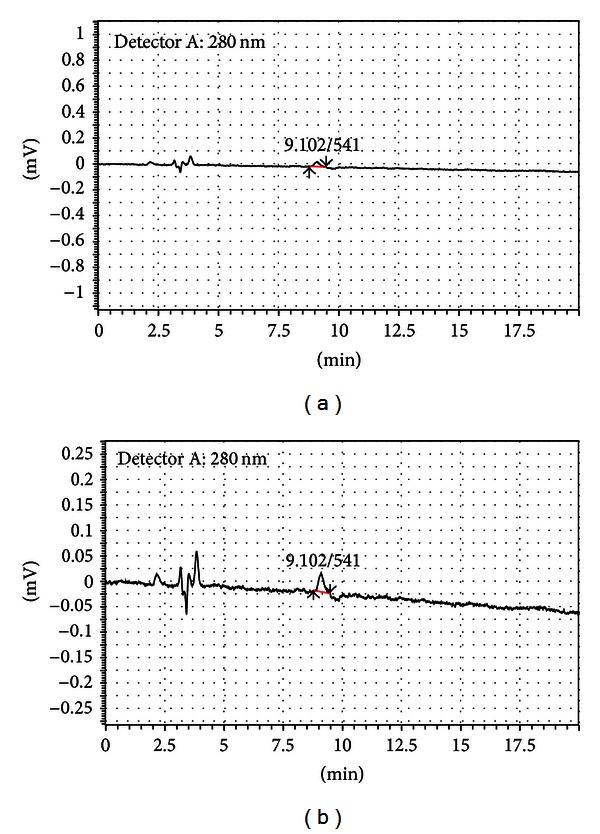
HPLC chromatograms for a 10 *μ*L injection of a 0.06 *μ*g/mL FES standard in acetonitrile. (a) is UV absorbance at 280 nm showing FES sensitivity and mobile phase impurities at this wavelength. (b) is an expanded scale of the UV absorbance at 280 nm (near the lambda max for FES). The retention time of the FES is 9.10 minutes for UV.

**Figure 6:**
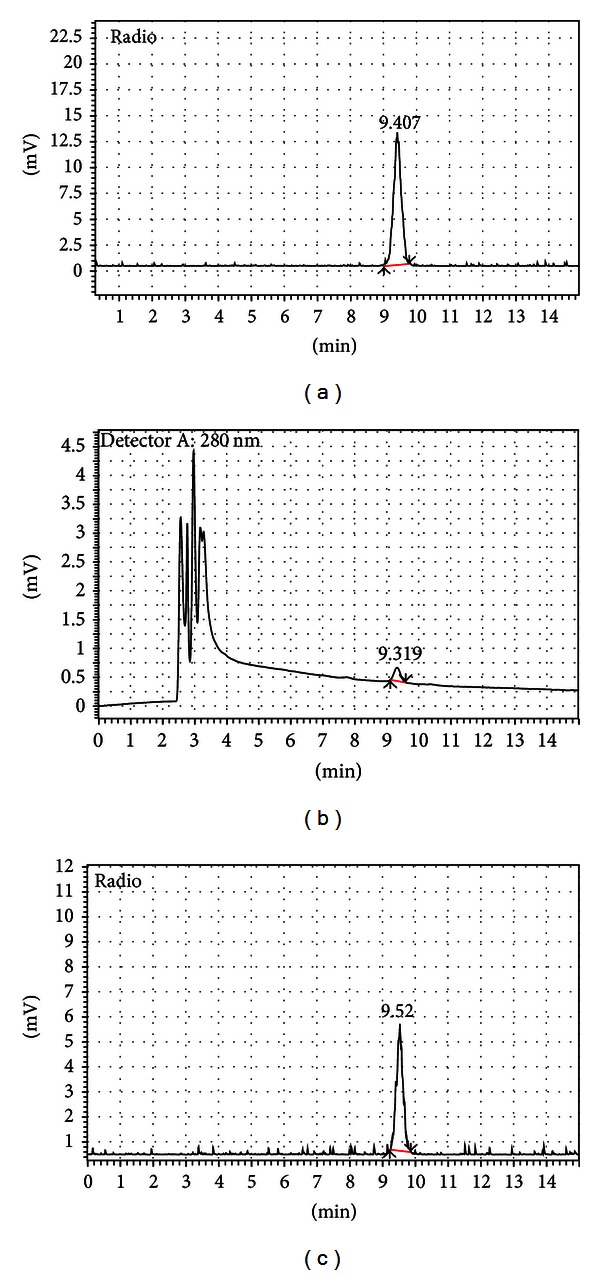
Typical HPLC chromatogram of [^18^F]FES product. (a) showed radiation peak of the [^18^F]FES product without coinjection of authentic standard. (b) showed UV absorbance at 280 nm of [^18^F]FES product along with coinjected authentic standard FES. (c) showed radiation peak of the co-injection. There is tubing between the UV and radiation detectors so that the FES is eluted at ~9.3 min for UV and ~9.5 min for radiation. UV peaks before 4 min are the UV absorption of formulation buffer of the product and acetonitrile solvent for authentic standard.

**Figure 7:**
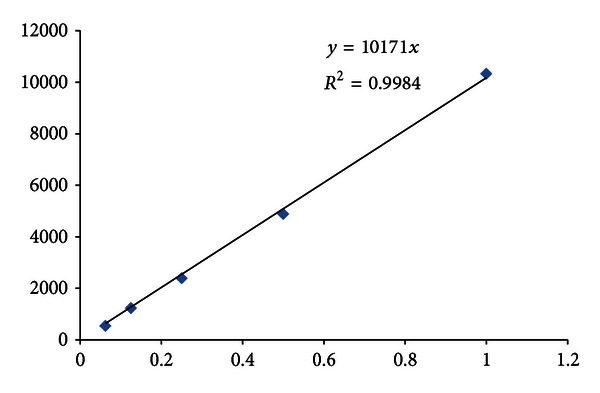
Standard calibration curve using FES authentic standard compound (conc. versus absorbance). *x*-axis represents the concentration of the authentic standard in *μ*g/mL (0.06 to 1.0 *μ*g/mL) and *y*-axis represents the absorbance (peak area).

Analytical HPLC is one of the most important QC experiments done to determine the quality of the injectable radiotracer, including the chemical and radiochemical purity, its identity, and the amount of impurities present in the final dose. The product is identified by co-injection of the radioactive drug with an authentic standard (Figures 5 and 6). During HPLC, [^18^F]FES is baseline separated from the other radioactive products and UV-absorbing compounds. Radiochemical purity of the product is >97% (Table 1). Typically, the final concentration of the [^19^F]FES (UV absorbance) in the [^18^F]FES product to be tested is below 5 *μ*g/dose (~12 mCi). Although FES is a weakly UV-absorbing compound, our analytical HPLC system is sensitive enough to detect less than 0.06 *μ*g/mL (Figure 5) of authentic FES standard, from which to develop a standard calibration curve (concentration versus absorbance, Figure 7). 

 Determination of the amount of FES drug (amount determined by UV) per radioactive dose (~12 mCi) is another way to express SA of the product. Though the SA is not a product releasing criterion, our simple calculation using *μ*g/dose shows that the SA of the final product is ~3500 ± 1500 Ci/mM (*n* = 3), which is high enough for medical use [16]. A recent clinical study (~240 patients) shows that the SA has no significant effect on tumor uptake of [^18^F] FES (expressed as standardized uptake value (SUV)) [16]. The SUV of [^18^F]FES over the range of SA values from 500–18000 Ci/mM is almost same. The body mass index (BMI) of the patients is one of the main factors that influence the ^18^F[FES] uptake [16].

Recently, it has been reported [17, 18] that the use of QMA cartridge-trapped ^18^F-fluoride in radiosynthesis reduces the SA of the tracer in comparison to the untrapped no-carrier-added ^18^F. SA between products produced by using QMA-trapped and QMA-untrapped ^18^F has not been compared in our production site. The QMA-trapped ^18^F obtained from Cardinal Health works very well. The amount of FES per dose is ~1.2 ± 0.5 *μ*g (average ± standard deviation for the three qualification runs) which is well below the maximum limit (≤5 *μ*g/dose, Table 1). 

#### 3.2.2. Impurities, Sterility, and Drug Product Stability

The total allowable, column-retained (after 4.0 min) UV-absorbing contaminants (peaks on the chromatogram) in the [^18^F]FES product must be ≤5 *μ*g in the injected dose, which assumes that the impurities have the same molar absorption coefficient for quantifying the impurity peaks. In the qualification runs, two or three impurity peaks were seen with retention times of 7.0 to 9.0 min. The retention time and peak intensities of the impurities are not exactly the same in all batches. They vary slightly from one batch to another. But, in all the cases, the overall impurity levels are well below the 5 *µ*g/dose limit. These impurities total to ~2.6 ± 1.0 *μ*g (*n* = 3).

The [^18^F]FES organic precursor (MMSE) does not elute from the HPLC column with the analytical mobile phase but elutes at ~8.1 min from the FES analytical column (Gemini C18) when the mobile phase is 80% methanol : 20% water (v : v). No precursor is expected in the final product. The acid hydrolysis should remove the sulfate- and the methylmethoxy-protecting groups and leave the major synthetic byproduct, estradiol. Any residual precursor, or sulfate- and methylmethoxy-protecting groups that were left, would not elute with the FES product when using the 50% ethanol preparative mobile phase. Other impurities that can be expected are K2.2.2 and residual solvents. K2.2.2 does not absorb UV light at 280 nm. K2.2.2 was assayed using a chemical spot test [14], which showed a level under 50 *μ*g/mL. Residual solvent levels (acetonitrile and acetone) were determined by GC using a standard calibration curve and were always below the limits stated in the USP recommendations. 

A conventional sterility testing requires ~14 days of time. For this reason, PET drug product ([^18^F]FES) was collected through a 0.22 *μ*m sterile filter and filter integrity was tested to make sure the product was likely to be pyrogen-free. If the 0.22 *μ*m filter used to filter the final product fails the integrity test both initially and upon rewetting of the filter then the final product will be resterilized using a new sterile filter.

The long-term radiochemical stability of [^18^F]FES in its final formulation at room temperature was determined using analytical HPLC. The final solution did not show any signs of decomposition up to 10 hrs. There was no increase of radiochemical or chemical impurities.

## 4. Conclusions

In this work, we present an automated, reliable, and reproducible radiosynthesis using the E & Z radiomodular synthesizer for the routine production of clinical-grade [^18^F]FES for medical use. Furthermore, the flexibility of this customizable automated synthesizer allows us to develop a rapid and straightforward automated synthetic and purification procedures to obtain [^18^F]FES with very high radiochemical yield (40.0 ± 5.0%) within just 80 min. Introduction of a simple two-step nonevaporative hydrolysis technique dramatically improved the overall yield and quality of the tracer. 

The QC results show that the product met all of the criteria of USP-grade radiopharmaceuticals as per FDA guidelines. This tracer will be used as a surrogate PET biomarker for the evaluation of endoxifen in ER+ breast cancer patients. 

## Figures and Tables

**Table 1:** QC results of three batches (FES-MBR-01, FES-MBR-02, and FES-MBR-03).

Quality control test	Description	Acceptance criteria	FES-MBR-01	FES-MBR-02	FES-MBR-03
Particulates and color	Visual inspection for color and particulates	Clear and colorless	Clear	Clear	Clear
Filter integrity	Bubble point test	>50 PSI	55	60	55
pH	pH as per USP 〈791〉 pH	pH must be between 6 and 8	7	7	7
Residual Kryptofix [2.2.2]^a^	Color spot test	<50 *μ*g/mL Kryptofix [2.2.2] by comparison with standard	<50 *μ*g/mL	<50 *μ*g/mL	<50 *μ*g/mL
Radiochemical purity		Radiochemical purity > 95%	97.8	99.7	99.9
FES (*μ*g per 12 mCi dose)	HPLC^b^, consistent with guidelines of USP 〈621〉	≤5 *μ*g per dose	0.72	1.68	1.58
Other UV impurities *μ*g/mL (*μ*g/dose)		≤5 *μ*g per dose	0.30 (3.6)	<0.06 (<1.7)	<0.06 (<1.7)
Radiochemical purity	TLC	*R*_*f*_ > 0.5 and purity ≥ 95%	0.8 and 98.6	0.8 and 96.6	0.7 and 96.9
Residual solvent levels^c^	Gas chromatography	Acetone < 5,000 ppm Acetonitrile < 400 ppm	<3125 <250	<3125 <250	<3125 <250
Radionuclidic purity	Half-life determination	105–120 minutes	110	111	110
Bacterial endotoxin levels	Limulus amoebocyte lysate (LAL) by gel clot or PTS	<175 EU per dose	<2	<2	<2
*Sterility test (14 days)	USP sterility test (USP 〈71〉)	No growth	No growth	No growth	No growth

^a^Kryptofix (K2.2.2) content was determined with the TLC spot test as per USP guidelines (TLC solvent: 9 : 1 solution of methanol and 30% ammonium hydroxide (v : v); TLC material: silica 60, *R*_*f*_ = 0.1; Kryptofix standard solution for visual testing: 50 *μ*g/mL; TLC development: iodine chamber).

^b^Phenomenex Gemini C_18_ reversed-phase HPLC column with a mobile phase of 50% ethanol : 50% water (v : v). The column flow rate is 0.5 mL/min.

^c^Acetone < 5,000 ppm and acetonitrile < 400 ppm as per USP 〈467〉 Organic Volatile Impurities.

*Not a release criterion.
